# Identification and characterization of plant Haspin kinase as a histone H3 threonine kinase

**DOI:** 10.1186/1471-2229-11-73

**Published:** 2011-04-28

**Authors:** Daisuke Kurihara, Sachihiro Matsunaga, Tomohiro Omura, Tetsuya Higashiyama, Kiichi Fukui

**Affiliations:** 1 Division of Biological Science, Graduate School of Science, Nagoya University, Furo-cho, Chikusa-ku, Nagoya, Aichi 464-8602, Japan; 2 JST ERATO Higashiyama Live-Holonics Project, Furo-cho, Chikusa-ku, Nagoya, Aichi 464-8602, Japan; 3 Department of Biotechnology, Graduate School of Engineering, Osaka University, 2-1 Yamadaoka, Suita, Osaka 565-0871, Japan; 4 Department of Applied Biological Science, Faculty of Science and Technology, Tokyo University of Science, 2641 Yamazaki, Noda, Chiba 278-8510, Japan

## Abstract

**Background:**

Haspin kinases are mitotic kinases that are well-conserved from yeast to human. Human Haspin is a histone H3 Thr3 kinase that has important roles in chromosome cohesion during mitosis. Moreover, phosphorylation of histone H3 at Thr3 by Haspin in fission yeast, *Xenopus*, and human is required for accumulation of Aurora B on the centromere, and the subsequent activation of Aurora B kinase activity for accurate chromosome alignment and segregation. Although extensive analyses of Haspin have been carried out in yeast and animals, the function of Haspin in organogenesis remains unclear.

**Results:**

Here, we identified a Haspin kinase, designated AtHaspin, in *Arabidopsis thaliana*. The purified AtHaspin phosphorylated histone H3 at both Thr3 and Thr11 *in vitro*. Live imaging of AtHaspin-tdTomato and GFP-α-tubulin in BY-2 cells showed that AtHaspin-tdTomato localized on chromosomes during prometaphase and metaphase, and around the cell plate during cytokinesis. This localization of AtHaspin overlapped with that of phosphorylated Thr3 and Thr11 of histone H3 in BY-2 cells. AtHaspin-GFP driven by the native promoter was expressed in root meristems, shoot meristems, floral meristems, and throughout the whole embryo at stages of high cell division. Overexpression of a kinase domain mutant of AtHaspin decreased the size of the root meristem, which delayed root growth.

**Conclusions:**

Our results indicated that the Haspin kinase is a histone H3 threonine kinase in *A. thaliana*. AtHaspin phosphorylated histone H3 at both Thr3 and Thr11 *in vitro*. The expression and dominant-negative analysis showed that AtHaspin may have a role in mitotic cell division during plant growth. Further analysis of coordinated mechanisms involving Haspin and Aurora kinases will shed new light on the regulation of chromosome segregation in cell division during plant growth and development.

## Background

The mitotic phase, which comprises mitosis and cytokinesis, is a fundamental process for faithful transmission of genetic information from one cell generation to the next. The main purpose of mitosis is to segregate sister chromatids into two daughter cells. The regulation of mitotic progression relies mainly on two post-translational mechanisms; protein phosphorylation and proteolysis. Cell division is regulated by mitotic kinases, such as the cyclin-dependent kinase 1 (CDK1), the Polo family, the NIMA (never in mitosis A), and the Aurora family, as well as kinases implicated in mitotic checkpoints, mitotic exit and cytokinesis [1].

Post-translational modifications of core histones play a crucial role in chromatin structure and gene expression [2]. Although the N-terminal sequence and phosphorylations of histone H3 are highly conserved among eukaryotes, the distribution patterns of phosphorylated histone H3 on the chromosomes differ between animals and plants. In mammalian cells, H3S10ph begins to appear in pericentromeric regions from G2 phase, spreading along the chromosome periphery until metaphase, and then disappearing at late anaphase [3]. The phosphorylation pattern of H3S28 is similar to that of H3S10ph during mitosis [4,5]. Because the spatial and temporal patterns of H3S10ph and H3S28ph are consistent with chromosome condensation and decondensation, it is thought that H3S10ph and H3S28ph have a crucial role in chromosome condensation in animals. In contrast, H3S10ph and H3S28ph occur in the pericentromeric regions--not along the whole chromosome--from prophase to anaphase in plants [6-8]. These distribution patterns suggest that H3S10ph and H3S28ph play a crucial role in cohesion and segregation of sister chromatids [9]. In plants, AtAUR3 (*Arabidopsis thaliana *Aurora kinase3) phosphorylates histone H3 at Ser10 and Ser28 *in vitro *[8,10,11]. Inhibition of Aurora kinase by Hesperadin treatment prevents H3S10ph and H3S28ph in tobacco BY-2 cells and H3S10ph in *Arabidopsis *suspension cells [8,12]. Thus, Aurora kinases phosphorylate histone H3 at Ser10 and Ser28 in plants.

H3T3 and H3T11 are also phosphorylated, but their distribution patterns differ from those of H3S10ph and H3S28ph during mitosis. In mammalian cells, H3T3ph and H3T11ph occur preferentially at the centromere from prophase to anaphase [13,14]. In contrast, H3T3ph and H3T11ph are distributed along the entire length of the chromosome in plants [15,16]. Aurora kinases phosphorylate histone H3 at Ser10 and Ser28, but the kinase responsible for H3T3ph and H3T11ph is yet to be identified in plants.

Haspin (haploid germ cell-specific nuclear protein kinase) was first identified as a testis-specific gene in mice [17,18]. Although Haspin mRNA levels were highest in the testis, lower levels of Haspin mRNA were detected in other organs, suggesting that expression of Haspin is not truly specific to haploid germ cells [19]. Human Haspin could phosphorylate histone H3 at Thr3 and was involved in chromosome congression during mitosis [13]. The centromeric localization of H3T3ph and the Haspin-knockdown phenotype in human cells indicated that Haspin is required for maintenance of centromeric cohesion during mitosis [20,21]. Recently, three studies on *Saccharomyces cerevisiae*, *Xenopus*, and human revealed the novel cascade leading to the recruitment of mitotic kinases to the centromere [22-24]. In *S. cerevisiae*, Haspin interacts with cohesin, and the cohesin-associated Haspin phosphorylates histone H3 at Thr3 on the inner centromere [22]. The phosphorylated H3T3 then binds the chromosomal passenger complex (CPC) containing Aurora B, thereby recruiting CPC to the inner centromere [22-24]. Thus, CPC functions in determining the correct kinetochore-microtubule attachment for accurate chromosome alignment and segregation, and this function is regulated via H3 phosphorylation on the inner centromere.

Analyses of Haspin were first carried out in yeast and animals, and although it is clear that this protein has roles in mitosis and cell division, the function of Haspin in organogenesis remains unclear. In this study, we identified *A. thaliana *Haspin, characterized its kinase activity, and determined its localization during mitosis. Expression of a kinase domain mutant of AtHaspin inhibited root growth, suggesting that Haspin is involved in cell division during mitosis.

## Results

### Haspin candidate gene in *Arabidopsis thaliana*

**Figure 1:**
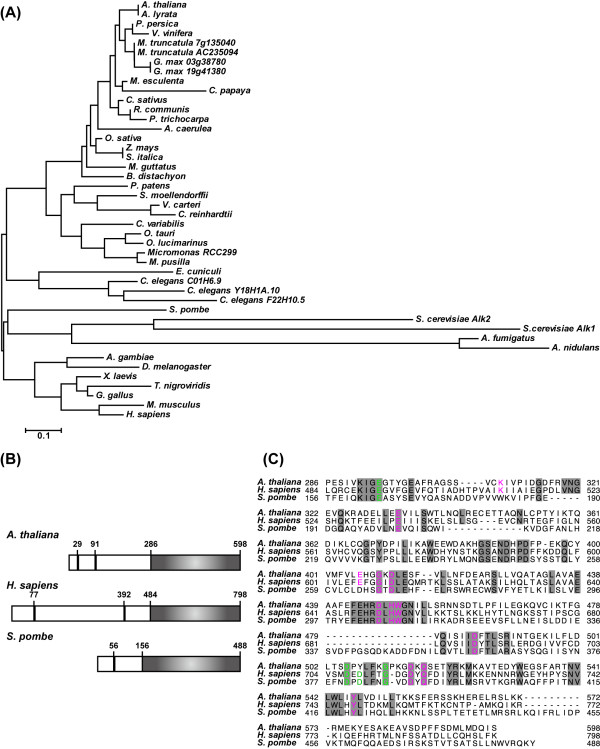
**Multiple alignment of Haspin kinases in the kinase domain**. (A) Kinase domains from *Anopheles gambiae *(EAA05110), *Aquilegia caerulea *(AcoGoldSmith_v1.025146m), *Arabidopsis rylata *(XP_002889750), *Arabidopsis thaliana *(NP_172416), *Aspergillus fumigatus *(XP_751829), *Aspergillus nidulans *(XP_659658), *Brachypodium distachyon *(Bradi1g20070.1), *Caenorhabditis elegans *F22H10.5 (NP_510696), *C. elegans *C01H8.9 (NP_492043), *C. elegans *Y18H1A.10 (NP_490768), *Carica papaya *(evm.model.supercontig_48.218), *Chlamydomonas reinhardtii *(XP_001699957), *Chlorella variabilis *(EFN57276), *Cucumis sativus *(Cucsa.050880.1), *Drosophila melanogaster *(P83103), *Encephalitozoon cuniculi *(NP_597598), *Gallus gallus *(XP_425408), *Glycine max *03g38780 (Glyma03g38780.1), *G. max *19g41380 (Glyma19g41380.1), *Homo sapiens *(AAH47457), *Manihot esculenta *(cassava4.1_028012m), *Medicago truncatula *(AC235094_20.1), *M. truncatula *(Medtr7g135040.1), *Mimulus guttatus *(mgv1a027116m), *Micromonas pusilla *(XP_003057374), *Micromonas RCC299 *(XP_002502153), *Mus musculus *(NP_034483), *Physcomitrella patens *(XP_001777245), *Populus trichocarpa *(XP_002329997), *Prunus persica *(ppa015455m), *Oryza sativa *(BAC16406), *Ostreococcus lucimarinus *(XP_001417826), *Ostreococcus tauri *(XP_003079484), *Ricinus communis *(XP_002512572), *Saccharomyces cerevisiae *Ybl009wp (NP_009544), *S. cerevisiae *ALK-1 (CAA61012), *Schizosaccharomyces pombe *(CAB16874), *Selaginella moellendorffii *(XP_002986955), *Setaria italica *(SiPROV006697m), *Tetraodon nigroviridis *(CAF92724), *Vitis vinfera *(XP_002276683), *Volvox carteri *(XP_002952488), *Xenopus laevis *(TC388096), and *Zea mays *(NP_001149827). Accession numbers from the DNA Data Bank of Japan (DDBJ) or transcript names from the genome database (Phytozome v6.0) are given in parentheses. (B) Amino acid structure of Haspin proteins from *A. thaliana*, *H. sapiens*, and *S. pombe*. Black boxes show NLSs (nuclear localization signals) predicted by the PSORT algorithm (http://psort.nibb.ac.jp/form.html). Gray box indicates kinase domain. (C) Multiple alignment of kinase domain of AtHaspin, human Haspin, and fission yeast Haspin. Missing residues are shown as dashes, identical amino acids are shaded in gray, and residues of ATP/Mg^2+ ^ion-binding sites are shown in magenta. Important residues for histone H3 phosphorylation in catalytic cleft are shown in green.

Genes encoding Haspin homologs have been identified in a wide variety of eukaryotes including vertebrates, invertebrates, plants, and fungi, but not in prokaryotes and archaea [25] (Figure 1A). Except for *Caenorhabditis elegans *and *S. cerevisiae*, most organisms have one Haspin kinase gene. In a BLAST search of the *A. thaliana *genome, one Haspin candidate gene showed high similarity (BLAST score = 196, E-value = 3E^-50^) to human Haspin kinase in the kinase domain. The second hit gene showed lower similarity (BLAST score = 46.6, E-value = 4E^-5^). One Haspin candidate gene has been identified in some plant species, including ferns, mosses, and algae (Figure 1A). Although there were two putative genes identified in *Glycine max *and *Medicago truncatula*, the synteny analysis from Phytozome [26] suggested that these genes were duplicated. In the *A. thaliana *genome, the putative Haspin gene (At1g09450) is designated as AtHaspin (*A. thaliana *Haspin-related gene). The C-terminal regions of Haspin proteins have a conserved kinase domain [27] (Figure 1B). The amino acid sequence of AtHaspin cDNA showed 38% similarity with human Haspin in the kinase domain. Recently, the crystal structure of the kinase domain of human Haspin was solved [28,29]. Although AtHaspin showed low similarity to human Haspin across the entire kinase domain, the residues that act as ATP and Mg^2+ ^ion-binding sites were conserved between human and *A. thaliana *(Figure 1C). These data suggested that the mitotic kinase function of Haspin may be conserved in plants.

### AtHaspin phosphorylates histone H3 at Thr3 and Thr11 *in vitro*

**Figure 2:**
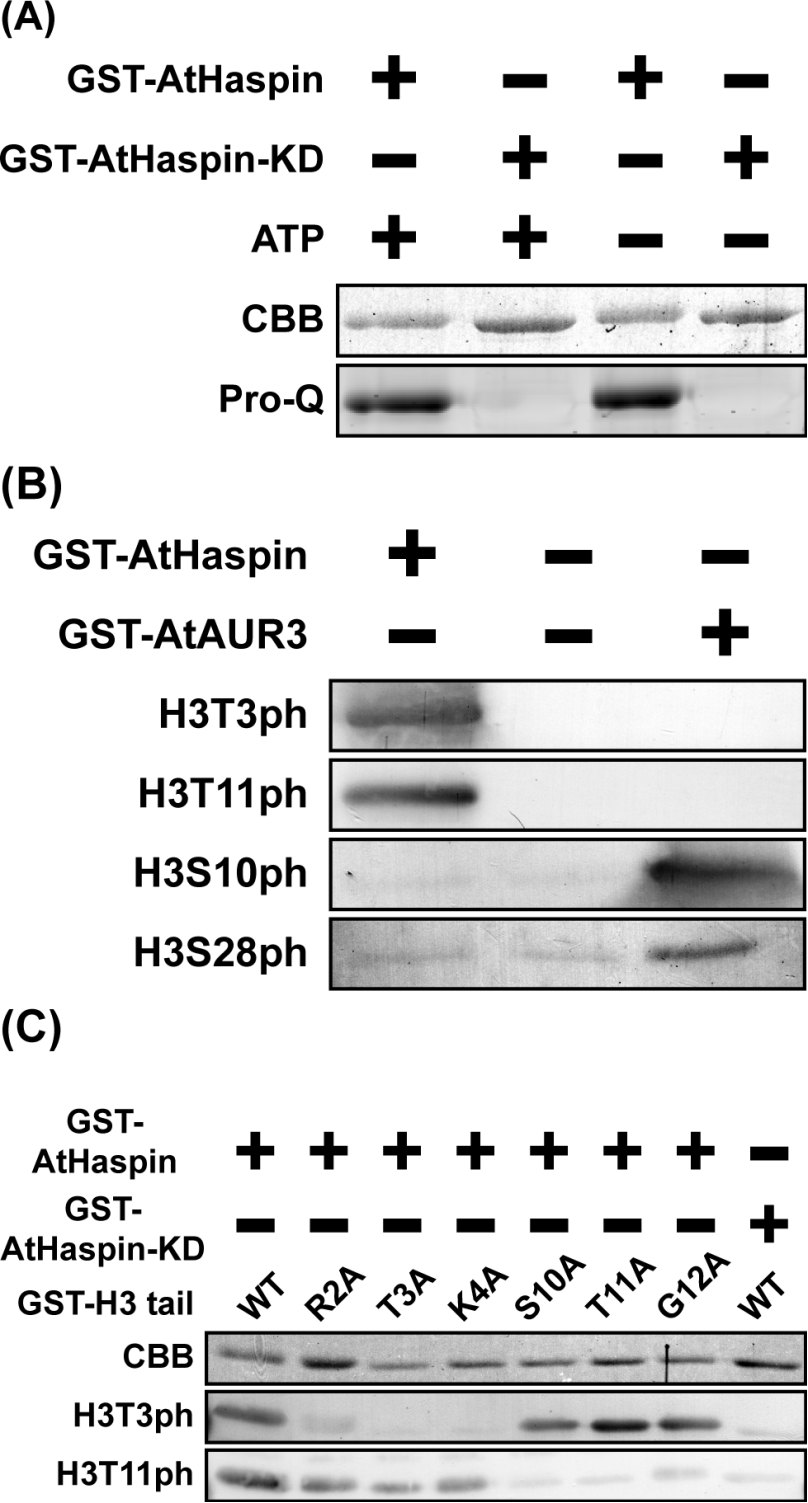
**GST-AtHaspin phosphorylates histone H3 at Thr3 and Thr11 *in vitro***. (A) GST-AtHaspin and GST-AtHaspin-KD were incubated with or without ATP, and phosphorylated proteins were stained with ProQ Diamond Phosphoprotein stain. (B) GST-AtHaspin and GST-AtAUR3 were incubated with GST-H3 tail (left and right lanes). Negative control: GST-H3 tail only (middle lane). Phosphorylated GST-H3 tail was immunostained using anti-H3T3ph, H3T11ph, H3S10ph, and H3S28ph antibodies. (C) GST-AtHaspin and GST-AtHaspin-KD were incubated with GST-H3 tails or mutants as substrates. Phosphorylated GST-H3 tails were immunostained with anti-H3T3ph and anti-H3T11ph antibodies.

The human Haspin protein K511A, which contains a mutation of a single conserved lysine residue that is important for ATP binding, has no kinase activity [13]. To examine whether purified GST-AtHaspin has kinase activity, an *in vitro *kinase assay was performed using purified GST-AtHaspin and GST-AtHaspin KD (kinase dead) (K309A) with or without ATP. Phosphorylated proteins were detected by ProQ Diamond Phosphoprotein Stain. As expected, GST-AtHaspin-KD was not autophosphorylated even in the presence of ATP (Figure 2A, second lane). However, GST-AtHaspin was autophosphorylated in the presence or absence of ATP (Figure 2A, first and third lanes). This result indicated that autophosphorylation of AtHaspin was not dependent on addition of ATP, and that this lysine residue is also required for autophosphorylation of AtHaspin. This result also suggested that GST-AtHaspin was autophosphorylated during production in *Escherichia coli*.

The only known substrate of Haspin is the Thr3 of histone H3 [13]. To determine whether AtHaspin is a histone H3 Thr3 kinase, we carried out an *in vitro *kinase assay using purified GST-AtHaspin, a positive control (GST-AtAUR3), and plant histone H3 as the substrate. The positive control, GST-AtAUR3, is a histone H3 Ser10 and Ser28 kinase [8]. GST-AtAUR3 phosphorylated histone H3 at Ser10 and Ser28, while GST-AtHaspin phosphorylated histone H3 at Thr3 *in vitro*. Surprisingly, GST-AtHaspin also phosphorylated histone H3 at Thr11 *in vitro *(Figure 2B).

To confirm the specificity of antibodies against H3T3ph and H3T11ph, an *in vitro *kinase assay was performed with purified GST-histone H3 tail proteins containing mutations at Thr3, Thr11, and at several residues adjacent to them (Arg2, Lys4, Ser10, and Gly12). Using anti-H3T3ph antibodies, bands were detected in the case of normal histone H3 and S10A, T11A, and G12A mutants, but not in the case of R2A, T3A, or K4A mutants (Figure 2C). Using anti-H3T11ph antibodies, bands were detected in the case of R2A, T3A, and K4A mutants, but not in the case of S10A, T11A, or G12A mutants (Figure 2C). GST-AtHaspin-KD (kinase domain mutant; K309A) had no kinase activity towards H3 at Thr3 and Thr11 *in vitro*. These results indicated that the AtHaspin kinase phosphorylates histone H3 at Thr3 and Thr11 *in vitro*.

### Subcellular localization of AtHaspin in BY-2 cells

**Figure 3:**
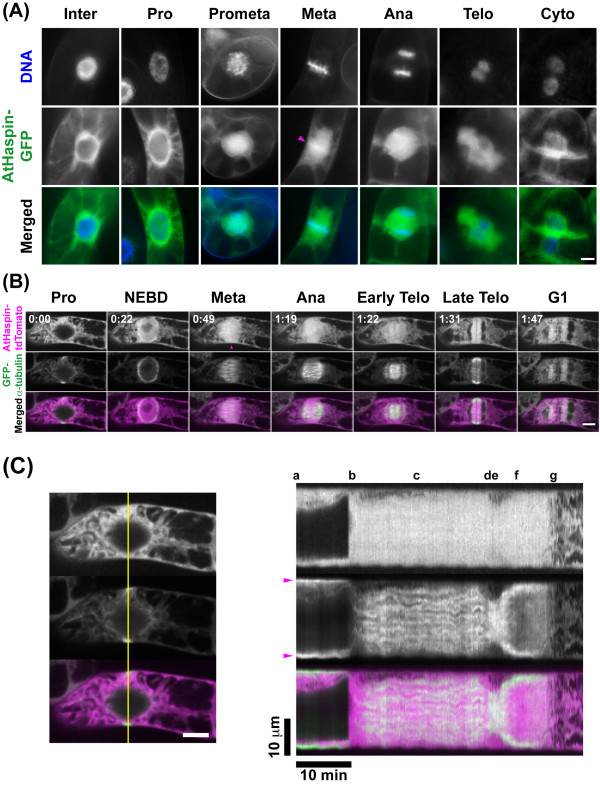
**Subcellular localization of AtHaspin in living tobacco BY-2 cells**. (A) DNA staining with Hoechst 33342 (top row), GFP fluorescence (middle row), and merged images (bottom row) showing DNA (blue) and GFP (green). Magenta arrowhead indicates fluorescent signal on chromosomes. (B) Live cell imaging was carried out in BY-2 cells expressing GFP-α-tubulin after more than 48-h induction of AtHaspin-tdTomato with 10 μM 17-β-estradiol. Merged images show AtHaspin-tdTomato (magenta) and GFP-α-tubulin (green). Magenta arrowhead indicates fluorescent signal on chromosomes. Numbers indicate time of observation (h: min) in additional file 1. (C) Kymographs representing fluorescence on yellow lines in left column. Arrows indicate PPB. Letters indicate mitotic stages as shown in (B). Scale bars: 10 μm (left), 10 min (bottom).

To analyze the subcellular localization of AtHaspin during cell division, we transformed *Nicotiana tabacum *cv. Bright Yellow-2 (tobacco BY-2) cultured cells with GFP-fused AtHaspin and observed tobacco BY-2 cells stably expressing AtHaspin-GFP with DNA stained by Hoechst 33342 (Figure 3A). During interphase, AtHaspin was mainly localized in the cytoplasm and at the nuclear periphery. After nuclear envelope breakdown (NEBD), AtHaspin invaded the nuclear region. During metaphase, fluorescent signals of AtHaspin-GFP were also observed on the chromosome (Figure 3A, arrowhead). After metaphase, AtHaspin-GFP was localized with the phragmoplast from its initial formation at the center of the equatorial plane to its expansion towards the cell periphery as the cell cycle progressed.

To analyze the relationship between AtHaspin and microtubules, we observed transgenic BY-2 cells expressing GFP-α-tubulin and inducibly expressing AtHaspin-tdTomato. After NEBD, AtHaspin-tdTomato immediately invaded the nucleus, while α-tubulin remained at the nuclear periphery. During prometaphase and metaphase, microtubules organized the mitotic spindle, while AtHaspin-tdTomato was widely distributed over the spindle. AtHaspin-tdTomato signals were observed on the chromosomes aligned at the equatorial plate (Figure 3B, arrowhead). During anaphase, AtHaspin-tdTomato localized with the sister chromatids, and during telophase, it colocalized with the phragmoplast. As the phragmoplast expanded toward the cell periphery, AtHaspin was moved toward the cell periphery. However, the movement of AtHaspin-tdTomato differed from that of phragmoplast (Figure 3C, Additional file 1). At the onset of cell division in higher plants, the pre-prophase band (PPB), which is a dense band of cortical microtubules, begins to form at the future-cell-division plane. Until NEBD, AtHaspin-tdTomato was localized at the cytoplasm but not at the PPB (Figure 3C; a, arrowhead), and then AtHaspin-tdTomato dispersed after NEBD (Figure 3C; b and c). As the cell plate expanded during telophase, the phragmoplast was depolymerized in the center of the equatorial plate and repolymerized along the edge of the growing cell plate. Although the phragmoplast expanded toward the cell periphery, AtHaspin-tdTomato remained around the cell plate until the end of telophase (Figure 3C; f). Considering the dynamics of AtHaspin-tdTomato, AtHaspin could not be directly involved in the regulation of the dynamics of microtubules during mitosis.

**Figure 4:**
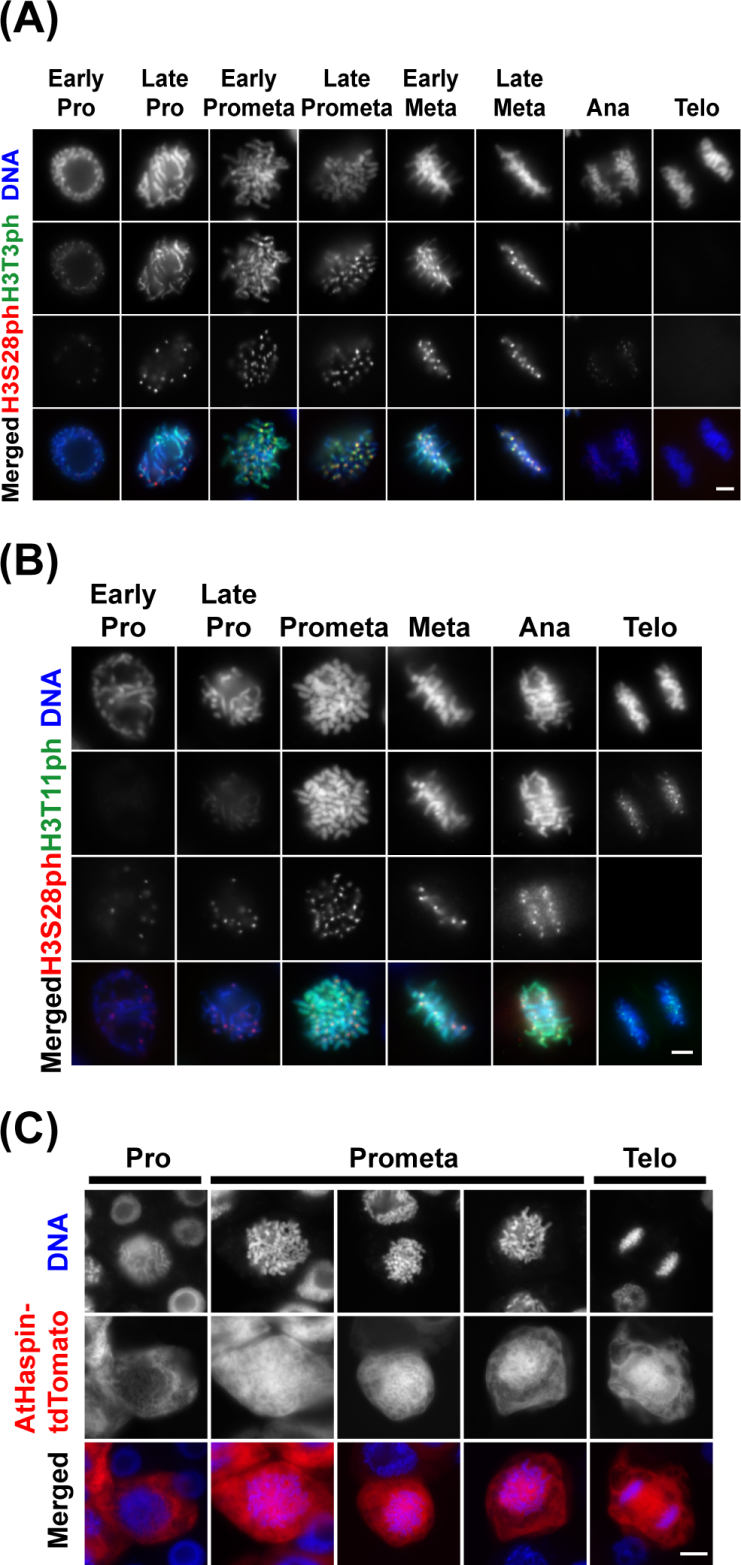
**Phosphorylation of histone H3 at Thr3 and Thr11 *in vivo***. (A, B) Phosphorylation of histone H3 at Thr3 and Thr11 during cell cycle. BY-2 cells immunostained using anti-H3T3ph (A), anti-H3T11ph (B), or anti-H3S28ph antibodies. DNA was stained with DAPI. Merged images of DNA (blue), H3S10ph (red) and H3S28ph (green) are shown in color. Scale bars: 10 μm. (C) After 48-h induction with 10 μM 17-β-estradiol, BY-2 cells inducibly expressing AtHaspin-tdTomato were fixed with 4% (w/v) paraformaldehyde for 20 min. DNA was stained with DAPI. Merged images of DNA (blue) and AtHaspin-tdTomato (red) are shown in color. Scale bars: 10 μm.

To reveal whether AtHaspin phosphorylates histone H3 at Thr3 and Thr11 *in vivo*, we performed indirect immunofluorescence using anti-H3T3ph, H3T11ph, and H3S28ph antibodies in BY-2 cells (Figures 4A and 4B). During interphase, no signals of H3T3ph, H3T11ph, and H3S28ph were observed. The H3T3ph signal was first detected on chromosomes in early prophase before NEBD, whereas the H3T11ph signal was first detected on chromosomes in late prophase after NEBD. During late prophase, H3T3ph signals were observed along the chromosome, while H3S28ph signals were observed at pericentromeric regions. During prometaphase, the H3T3ph signals were stronger at pericentromeric regions than on the chromosome arms. Signals of H3T3ph increased at the pericentromeric region until late metaphase. In contrast, phosphorylated H3T11 was entirely localized on the chromosome from prometaphase to anaphase. Signals of H3T3ph disappeared after chromosome segregation during anaphase, while H3T11ph signals were still localized on the chromosome. A moderate-strength AtHaspin-tdTomato signal was observed on the chromosome during prometaphase and metaphase (Figures 3A and 3B, arrowhead). This localization of AtHaspin-tdTomato overlapped with those of phosphorylated H3T3 and H3T11 from after NEBD until anaphase in BY-2 cells (Figures 4A and 4B).

Then, we observed the localization of AtHaspin-tdTomato in fixed BY-2 cells with paraformaldehyde (Figure 4C). At early prophase before NEBD, AtHaspin-tdTomato was localized in the cytoplasm around the nucleus (Figure 4C; Pro). At prophase after NEBD, AtHaspin-tdTomato enveloped the entire chromosome (Figure 4C; Prometa). This localization also suggested that AtHaspin phosphorylates histone H3 at Thr3 and Thr11 during mitosis.

### Expression of AtHaspin in developing organs

**Figure 5:**
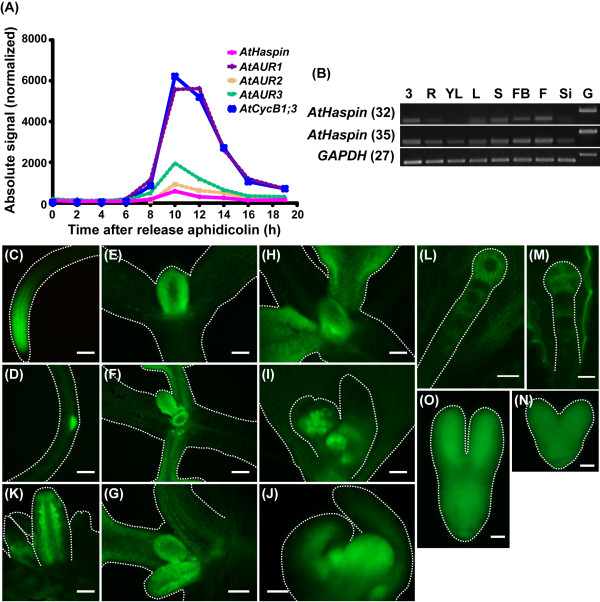
**Expression patterns of AtHaspin-GFP in *Arabidopsis***. (A) Expressions of *AtHaspin*, *AtAUR*s, and *AtCycB1;3 *during mitotic cell cycle in synchronized *Arabidopsis *cultured cells. Expression data were obtained from publicly available microarray data [30]. Figure shows expressions of genes after removal of the DNA synthesis inhibitor, aphidicolin. (B) Total RNA was extracted from 3-day-old seedlings (3), roots (R), young leaves (YL), leaves (L), stems (S), flower buds (FB), flowers (F), siliques (Si), and genomic DNA (G). Expression was monitored by RT-PCR. Number of PCR cycles is shown in parentheses after gene names. *GAPDH *was used as an internal control. (C-N) Expression of AtHaspin-GFP in root tip (C), lateral root (D), shoot meristem and leaf primordia (E-H), leaf primordia and first true leaves (F, G), leaf primordia and second true leaves (H), inflorescence meristem and floral meristem in cauline leaves (I), floral meristem (J), ovules in closed flowers (K), one-cell stage embryo (L), four-cell stage embryo (M), heart stage embryo (N), and torpedo stage embryo (O). Scale bars: 100 μm (C, D, F, H, I, J), 50 μm (E), 30 μm (G, K), 10 μm (L, M), and 20 μm (N, O).

According to the microarray data publically available at Genevestigator [30], *AtHaspin *is expressed at relatively high levels in the root tips and shoot apex. We plotted the RNA profiles using published microarray datasets [31]. Expression of *AtHaspin *was activated at 8 to 16 h after removal of the DNA synthesis inhibitor, aphidicolin (Figure 5A). This profile of *AtHaspin *expression was very similar to those of *AtAUR*s and mitotic *AtCycB1;3*, indicating that AtHaspin is a mitotic-specific kinase. RT-PCR analyses showed that AtHaspin was expressed in multiple tissues (Figure 5B). Although Haspin was first identified as a testis-specific gene in mice [17,18], this expression profile indicated that AtHaspin is not a reproduction-specific gene in *A. thaliana*. AtHaspin showed relatively high expression in flower buds and flowers with high cell division activity.

To investigate the expression patterns of AtHaspin in *Arabidopsis *plants, we produced transgenic plants expressing AtHaspin-GFP under the control of a native promoter region (the region 1672-bp upstream of the translation initiation codon of AtHaspin). AtHaspin-GFP was expressed in meristems and primordia of root tips, lateral roots (Figures 5C and 5D), the shoot apex, leaf (Figures 5E-5H), and flowers (Figures 5I-5K). Fluorescent signals of AtHaspin-GFP were observed in ovules (Figure 5J). During embryogenesis, AtHaspin-GFP was expressed in embryos and suspensors from the one-cell stage to the four-cell stage (Figures 5L and 5M). The expression of AtHaspin-GFP persisted in the torpedo-stage embryo (Figures 5N and 5O). Thus, the expression patterns of AtHaspin-GFP were strongly correlated with cell division during organ development.

To study the subcellular localization of AtHaspin in detail, we performed time-lapse analysis of AtHaspin-GFP in root tips and the embryo. At interphase, the fluorescent signals of AtHaspin-GFP were observed in the cytoplasm. During mitosis, AtHaspin-GFP was invaded the nucleus at prometaphase and expanded toward the cell periphery at cytokinesis (Additional files 2, 3 and 4). These localization patterns corresponded to those observed in BY-2 cells.

### Inducible AtHaspin-KD decreases meristem size in roots

**Figure 6:**
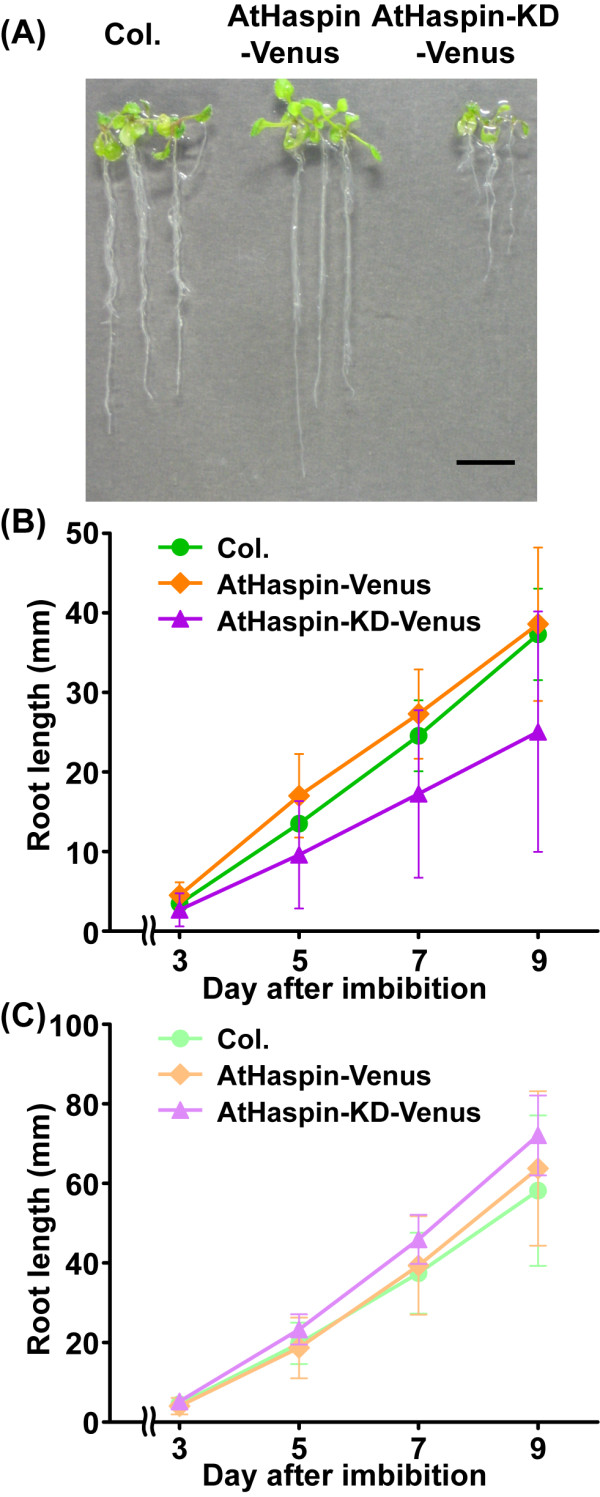
**Root growth defects in plants overexpressing AtHaspin-KD-Venus**. (A) At 11 days after imbibition, root growth was decreased in AtHaspin-KD-Venus overexpression plants compared with roots of *Col*. and AtHaspin-Venus overexpression plants. Scale bar: 10 μm. (B, C) Root length of transformants with inducible AtHaspin-Venus and AtHaspin-KD-Venus with (B) or without induction (C). Mean values ± standard error are shown (B; *n *≥ 15 plants, C; *n *= 10 plants).

**Figure 7:**
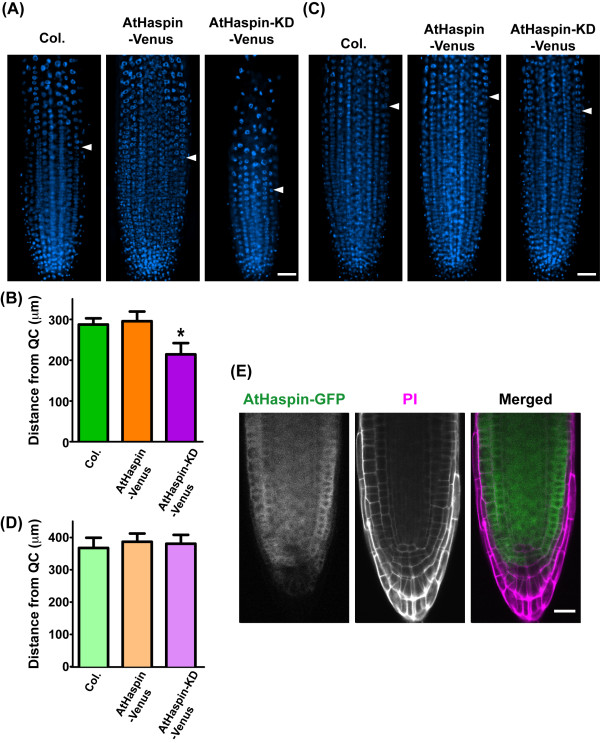
**Overexpression of AtHaspin-KD-Venus decreased the size of the root meristem**. (A, C) At 6 days after imbibition, DNA was stained with DAPI in transformants with inducible AtHaspin-Venus and AtHaspin-KD-Venus with (A) or without induction (C). Arrowheads indicate position of first endocycles in the epidermis. Position of first endoduplicated cells was estimated as described in materials and methods. Scale bar: 50 μm. (B, D) Distance of first endoduplicated cells from QC in epidermis and cell cortex in transformants with inducible AtHaspin-Venus and AtHaspin-KD-Venus with (B) or without induction (D). Mean values ± standard error are shown (*n *= 5 plants). One-way ANOVA with Bonferroni post-hoc test showed a significant difference between AtHaspin-KD-Venus overexpression plants and *Col*. (*p *< 0.001). (E) AtHaspin-GFP was expressed in root tips, except for QC and columella. Cell walls were stained with PI. Merged images show AtHaspin-GFP (green) and PI (magenta). Scale bar: 20 μm.

We searched T-DNA tagging lines to elucidate the function of AtHaspin; however, there were no AtHaspin knockout mutants. Considering the possibility that the loss-of function mutant of AtHaspin is embryonic lethal, we constructed a line with chemical-inducible overexpression of a kinase domain mutant of AtHaspin (AtHaspin-KD) using the estradiol-inducible XVE system [32]. When grown on vertically oriented plates with 10 μM 17-β-estradiol, AtHaspin-KD-Venus plants exhibited decreased primary root growth from 11 days after imbibition, compared with root growth of *Col*. and AtHaspin-Venus plants (Figures 6A and 6B). To investigate the effect of overexpression of AtHaspin-KD-Venus on root tip cells, 6-day-old roots were stained with 4',6-diamidino-2-phenylindole (DAPI) to observe the meristem by detecting the DNA ploidy level of the cells. The root meristem was smaller in AtHaspin-KD-Venus plants than in *Col*. plants, but the meristem was not affected in AtHaspin-Venus plants (Figures 7A and 7B). Moreover, most AtHaspin-KD-Venus plants showed abnormally oriented cell plates (4/5 plants, Additional file 5). These results suggested that misoriented cell divisions were responsible for the abnormal cell pattern in the root tips. These phenotypes were not observed in transgenic plants without induction of AtHaspin-Venus and AtHaspin-KD-Venus (Figure 6C, 7C, and 7D). AtHaspin-GFP was expressed in the meristem, but not in the quiescent center (QC) or the columella of root tips (Figure 7E). These results suggest that AtHaspin may have a role in mitotic cell division.

## Discussion

### AtHaspin phosphorylates histone H3 at both Thr3 and Thr11 *in vitro*

In this study, we identified a plant Haspin kinase in *A. thaliana*. Haspin kinases are conserved from yeast to human. Most organisms have one Haspin kinase gene, except for *C. elegans *and *S. cerevisiae*. Some plant species, including *A. thaliana*, have one Haspin candidate gene (Figure 1A). Even in the kinase domain, there is low homology (37%) between AtHaspin and human Haspin. However, the important residues for kinase activity, such as those associated with ATP/Mg^2+ ^ion-binding, are well-conserved in *A. thaliana *(Figure 1C).

As expected, GST-AtHaspin phosphorylated histone H3 at Thr3 *in vitro*. Surprisingly, GST-AtHaspin was also able to phosphorylate histone H3 at Thr11 *in vitro*. It has not been determined whether the Haspin of fission yeast or *Xenopus *can phosphorylate histone H3 at Thr11, however, the human Haspin phosphorylates histone H3 at Thr3, but not Thr11 *in vitro *[13,22,24]. In the case of Aurora kinase, because its consensus sequence is (RXS/T), AtAUR3 can phosphorylate histone H3 at Ser10 (A^7^R^8^K^9^S^10^) and Ser28 (A^25^R^26^K^27^S^28^) [33]. However, the amino acid sequence around Thr3 (A^1^R^2^T^3^K^4^) differs from that around Thr11 (K^9^S^10^T^11^G^12^). Point mutational analysis of human Haspin revealed the important residues for histone H3 phosphorylation in the catalytic cleft [29]. These residues are almost all conserved in *A. thaliana *(Figure 1C, green), with only one residue differing between human (Asp709) and *Arabidopsis *(Tyr507). The human Haspin mutant D709N shows reduced affinity for the histone H3 tail and impaired ability to phosphorylate H3 [29]. Although AtHaspin can phosphorylate histone H3, it is possible that its substrate specificity is wider than that of the human Haspin. In mammalian cells, the Dlk/ZIP kinase phosphorylates histone H3 at Thr11 *in vitro *[14], but there is no direct evidence that it phosphorylates histone H3 at Thr11 *in vivo*. However, the centromeric localization of Dlk/ZIP during mitosis suggests that this kinase is responsible for H3T11ph. The *A. thaliana *genome contains no Dlk/ZIP kinase orthologues, and thus, AtHaspin has an additional role as a H3 Thr11 kinase in *A. thaliana*.

### Phosphorylation of histone H3 at Thr3 and Thr11

Mitotic phosphorylation of histone H3 at Ser10, Ser28, Thr3, and Thr11 is highly conserved among eukaryotes. Although phosphorylation of histone H3 was first observed more than 30 years ago, the functions of this modification remain unclear [34]. There is an apparent correlation between H3S10ph and chromosome condensation during mitosis, suggesting that H3S10ph is important for chromosome structure. However, H3S10ph is not required for chromosome condensation in some species [35]. In animals, H3S10ph and H3S28ph occur along the entire chromosome, while H3T3ph and H3T11ph occur only at pericentromeric regions. The functions of H3T3ph and H3T11ph have not been characterized. In contrast to the case in animals, H3T3ph and H3T11ph are preferentially distributed along the entire chromosome in plants (Figures 4A and 4B). Thus, the localization and timing of histone H3 Ser and Thr phosphorylation during mitosis differ between plants and animals. However, the fact that Haspin and Aurora kinases are responsible for histone H3 phosphorylations in plants and animals suggests that the functions of histone H3 phosphorylations are conserved among eukaryotes.

Fluorescent protein fused to AtHaspin (AtHaspin-FP) was localized in the cytoplasm during interphase until NEBD. The interphase localization of AtHaspin differs from that of human Haspin, which localizes in the nucleus during interphase [36]. After NEBD, AtHaspin-FP invaded into the nucleus and spread along the chromosomes and throughout the cytoplasm until metaphase. A strong AtHaspin-FP signal was observed on the chromosome during prometaphase and metaphase. These localization patterns of AtHaspin-FP were consistent with that of AtHaspin-GFP driven by the native promoter in *A. thaliana *(Additional files 2, 3 and 4). Although no contigs containing Haspin orthologues were found in the BY-2 EST database [37], AtHaspin shares 61% amino acid sequence identity in the kinase domain to a tomato Haspin orthologue from the Kazusa Tomato SBM Database [38], suggesting that the localization and dynamics of AtHaspin-FP reflect those of an endogenous Haspin protein in BY-2 cells.

This localization of AtHaspin-FP is consistent with that of phosphorylated H3T3 and H3T11 in BY-2 cells (Figures 3 and 4). The timing of phosphorylation and dephosphorylation of H3T3 and H3T11 are distinct during mitosis. The H3T3ph begins at early prophase before NEBD, while H3T11ph occurs at prophase after NEBD. Dephosphorylation of H3T3 occurs at anaphase, while that of H3T11 occurs in telophase. These results suggested that there are different phosphatases responsible for H3T3 and H3T11 dephosphorylation in plants. We cannot exclude the possibility that there is another histone H3 Thr3 and Thr11 kinase in addition to AtHaspin, because AtHaspin was in the cytoplasm, but H3T3ph signals were detected before NEBD. Another possibility is the specificity of the antibodies against H3T3ph and H3T11ph. The *in vitro *kinase assay revealed that the antibodies against H3T3ph and H3T11ph did not show cross-reactivity with other histone H3 phosphorylations (Figure 2B and 2C). However, we do not know whether these antibodies react with other histone modifications *in vivo*. An Aurora kinase inhibitor prevents both H3S10ph and H3S28ph, even though these two phosphorylation events occur at different times during mitosis in tobacco BY-2 cells [8]. Similarly, AtHaspin could phosphorylate histone H3 at Thr3 and Thr11 *in planta*.

### AtHaspin is involved in cell division

Because of the presence of the plant cell wall, cell division, including cytokinesis, is more complex in plants than in animals. A plant-specific cytoskeletal structure, the phragmoplast, is required for completion of cytokinesis in plants [39]. AtHaspin-tdTomato was localized at the phragmoplast after chromosome segregation. Although the phragmoplast expanded to the cell wall, AtHaspin-tdTomato remained around the cell plate (Figure 3C; f). These dynamics of AtHaspin-tdTomato localization and movement suggested that AtHaspin is associated with cell division.

Analyses of *Arabidopsis *plants expressing AtHaspin-GFP driven by its own promoter indicated that AtHaspin is expressed in root meristems, shoot meristems, floral meristems, and embryos with high cell-division activities. In addition, overexpression of AtHaspin-KD-Venus resulted in defects in root growth. Plant growth is regulated by cell division and cell expansion, and defects in these processes resulted in delayed root growth. Cell expansion is regulated by microtubule-associated proteins (MAPs) [40]. MAPs have roles in microtubule polymerization, depolymerization, bundling, and nucleation. However, no filamentous structures of fluorescent protein-fused AtHaspin were observed at interphase or during mitosis in *Arabidopsis *and BY-2 cells (Figure 3, Additional files 1, 2, 3 and 4). The expression of AtHaspin-GFP in the root meristem, except for QC and columella cells, indicated that AtHaspin may have a role in cell division during mitosis (Figure 7E). Furthermore, some abnormally oriented cells were observed in root rips of plants overexpressing AtHaspin-KD-Venus (Additional file 5). Although further analysis of down-regulation of AtHaspin is needed, these results suggested that AtHaspin-KD-Venus may inhibit cell division in a dominant negative manner.

## Conclusions

In this study, the Haspin kinase in *A. thaliana *was identified as a mitotic histone H3 threonine kinase. The expression and dominant-negative analysis showed that AtHaspin may have a role in cell division during mitosis. The functions of H3 threonine phosphorylation remain obscure in animals and plants. Recent studies have shown that H3T3ph by *S. cerevisiae*, *Xenopus *and human Haspin is required for the accumulation of Aurora B on the centromere, and for subsequent activation of Aurora B kinase activity [22-24]. Therefore, AtHaspin and AtAUR3 coordinately regulate cell division during mitosis via H3 phosphorylation. As previously described [11,41], homologues of CPC components (INCENP, Survivin, and Borealin/DasraB) could not be found in plants via sequence similarities. However, the CPC has an important role in proper chromosome segregation via regulation of Haspin and Aurora B in animals and yeast [22-24]. The functions and localizations of Haspin and Aurora kinases are partly conserved in *A. thaliana *[8,10-12,42], suggesting that functional analogues of CPC components exist in plants. Further analyses of the substrates of AtHaspin and their downregulating mechanisms will provide insights into the regulation of cell division during plant growth and development.

## Methods

### Plant materials

*A. thaliana *(ecotype Columbia) seeds were grown on plates containing half-strength Murashige and Skoog salts, Gamborg B5 vitamins, 0.05% MES-KOH (pH 5.8), and 1% agar in a growth chamber at 22°C under continuous light. Tobacco 'Bright Yellow-2' (BY-2) cells were maintained as previously described by Kurihara et al. [42].

### Cloning

A full-length cDNA for the candidate Haspin kinase (RAFL15-11-J01) was obtained from the RIKEN BioResource Center [43]. Site-directed mutation of AtHaspin for P454L was generated by PCR-based mutagenesis. For construction of the GST fusion protein, the cDNA of *AtHaspin *was cloned into the pDEST15 expression vector by Gateway technology (Invitrogen). Site-directed mutations of GST-AtHaspin for K309A and of the GST-H3 tail for R2A, T3A, K4A, S10A, T11A, and G12A were generated by PCR-based mutagenesis from the GST-AtHaspin and GST-H3 tail expression vectors, as described elsewhere [44].

*AtHaspin *cDNA was cloned into the spUC-GFP vector, which contains the CaMV 35S promoter. spUC-AtHaspin-GFP was digested and ligated with pEBis-kH2 as described by Fujimoto et al. [45].

For inducible expression constructs, *AtHaspin *cDNA was cloned into the spUC-tdTomato vector [42]. For Venus expression constructs, tdTomato was replaced by Venus from mVenus/pRSETB [46]. PCR fragments of AtHaspin-tdTomato (or Venus) were cloned into pX7-GFP [32].

For construction of the GFP expression vector driven by the *AtHaspin *native promoter, the promoter and genomic regions were amplified from *A. thaliana *genomic DNA. PCR products were cloned into pENTR using a pENTR/D-TOPO cloning kit (Invitrogen), and then into the pGWB4 expression vector by the LR reaction of the Gateway Cloning System (Invitrogen).

### *In vitro *kinase assay

The *in vitro *kinase assay was performed with purified GST-AtHaspin or GST-AtHaspin-KD, the GST-histone H3 tail, and mutants as substrates as previously described by Kurihara et al. [8].

### Imaging

Transformation of BY-2 cells and BY-GT16 cells [47] was carried out using *Agrobacterium*-mediated methods as described previously [42]. Stable transgenic BY-2 cells expressing AtHaspin-GFP were observed using a fluorescence microscope (IX-81; Olympus). For induction of AtHaspin-tdTomato, 10 μM 17-β-estradiol (Sigma), or 0.1% ethanol (control) was added to 2-day-old cells, and then cells were cultured for a further 48 h. The cells were observed using a fluorescence microscope (IX-81; Olympus) equipped with a Nipkow disk confocal unit (CSU-X1; Yokogawa Electric). Indirect immunofluorescence was performed as described previously [42].

The floral-dip method was used for *Agrobacterium*-mediated *Arabidopsis *transformation [48]. Transgenic *Arabidopsis *plants expressing AtHaspin-GFP driven by the native promoter were observed using an upright fluorescence microscope (BX-51; Olympus), or FluoView FV1000 (Olympus). Cell walls were stained with 10 μg/ml propidium iodide (PI).

### Root phenotype analysis

For induction of AtHaspin-Venus and AtHaspin-KD-Venus, 10 μM 17-β-estradiol (Sigma) was added to MS medium (pH 5.8, 1.5% agar) to induce expression during seed germination in sterile conditions. The root length of vertically grown seedlings was measured with MBF ImageJ software.

The size of root meristems was determined by the position of the first endoduplicated cells, which were identified on the basis of their DNA content. Roots were fixed with 4% paraformaldehyde and 0.1% Triton X-100 for 40 min and stained with CyStain staining solution containing DAPI (Partec) for 5 min. Images were acquired using the IX-81 fluorescence microscope with a 0.5-μm section at the Z axis. These 2D images were used to construct 3D deconvolution images using AutoQuant X (Media Cybernetics). Cell ploidy was calculated using Metamorph (Molecular Devices). When three cells with more than 4C DNA content were aligned continuously, the top cell was defined as the first endoduplicated cell. Cell wall patterning was analyzed using the mPS-PI method [49]. PI-staining images were acquired under a fluorescence microscope (IX-81, Olympus) equipped with a confocal scanning unit (CSU-X1, Yokogawa Electric). Statistical analyses (one-way ANOVA, followed by Bonferroni post-hoc test) were performed using GraphPad Prism version 5.04 for Windows (GraphPad Software).

## Abbreviations

ANOVA: analysis of variance; BLAST: basic local alignment search tool; BY-2: Bright Yellow-2; CPC: chromosomal passenger complex; DAPI: 4',6-diamidino-2-phenylindole; EST: expressed sequence tag; GFP: green fluorescent protein; GST: glutathione S-transferase; H3S10ph: phosphorylation of histone H3 at Ser10; H3S28ph: phosphorylation of histone H3 at Ser28; H3T3ph: phosphorylation of histone H3 at Thr3; H3T11ph: phosphorylation of histone H3 at Thr11; KD: kinase dead; MAP: microtubule-associated protein; NEBD: nuclear envelope breakdown; PFA: paraformaldehyde; PPB: pre-prophase band; QC: quiescent center.

## Authors' contributions

DK conceived and designed the study, carried out the experiments, performed the statistical analyses, and wrote the manuscript. SM participated in the design of the study, carried out the ploidy analysis and the observations of AtHaspin-KD overexpression in root tips, and revised the draft of the manuscript. TO carried out the phenotypic analyses of plants overexpressing AtHaspin-KD. TH and KF participated in the coordination of the study. All authors read and approved the final manuscript.

## Supplementary Material

Additional file 1 **Dynamics of AtHaspin-tdTomato and GFP-α-tubulin in tobacco BY-2 cells**. Dynamics of AtHaspin-tdTomato (magenta) and GFP-α-tubulin (green) from late G2 phase to early G1 phase in BY-2 cells after 48-h induction with 10 μM 17-β-estradiol. Images were acquired at 30-s intervals under a 40 × objective lens, and movie is displayed at 15 frames per second (fps). Numbers indicate time (h: min). Click here for file

Additional file 2 **Dynamics of AtHaspin-GFP in *Arabidopsis *root tips**. Images were acquired at 1-min intervals under a 40 × objective lens, and movie is displayed at 15 fps. Numbers indicate time (h: min). Arrowheads indicate mitotic cells. Click here for file

Additional file 3 **Dynamics of AtHaspin-GFP in *Arabidopsis *torpedo embryo**. Images were acquired at 1-min intervals under a 40 × objective lens, and movie is displayed at 10 fps. Numbers indicate time (h: min). Arrowheads indicate mitotic cells. Click here for file

Additional file 4 **Dynamics of AtHaspin-GFP in *Arabidopsis *root tips**. Images were acquired at 90-s intervals under a 100 × objective lens, and movie is displayed at 5 fps. Numbers indicate time (h: min). Arrowheads indicate mitotic cells. Click here for file

Additional file 5 **Abnormality of cell orientation in AtHaspin-KD plants**. At 6 days after imbibition, *Col*. plants and transformants with inducible AtHaspin-KD-Venus vectors with or without induction were analyzed using the mPS-PI method. In AtHaspin-KD plants, arrows indicate abnormalities in orientations of cell walls between transverse neighboring cells. Scale bar: 50 μm. Click here for file

## References

[B1] Nigg EA Mitotic kinases as regulators of cell division and its checkpoints Nat Rev Mol Cell Biol 2001 2 1 21 32 11413462 10.1038/35048096

[B2] Kouzarides T Chromatin modifications and their function Cell 2007 128 4 693 705 10.1016/j.cell.2007.02.005 17320507

[B3] Hendzel MJ Wei Y Mancini MA Van Hooser A Ranalli T Brinkley BR Bazett-Jones DP Allis CD Mitosis-specific phosphorylation of histone H3 initiates primarily within pericentromeric heterochromatin during G2 and spreads in an ordered fashion coincident with mitotic chromosome condensation Chromosoma 1997 106 6 348 360 10.1007/s004120050256 9362543

[B4] Goto H Tomono Y Ajiro K Kosako H Fujita M Sakurai M Okawa K Iwamatsu A Okigaki T Takahashi T Identification of a novel phosphorylation site on histone H3 coupled with mitotic chromosome condensation J Biol Chem 1999 274 36 25543 25549 10.1074/jbc.274.36.25543 10464286

[B5] Goto H Yasui Y Nigg EA Inagaki M Aurora-B phosphorylates Histone H3 at serine28 with regard to the mitotic chromosome condensation Genes Cells 2002 7 1 11 17 10.1046/j.1356-9597.2001.00498.x 11856369

[B6] Houben A Wako T Furushima-Shimogawara R Presting G Kunzel G Schubert II Fukui K Short communication: the cell cycle dependent phosphorylation of histone H3 is correlated with the condensation of plant mitotic chromosomes Plant J 1999 18 6 675 679 10.1046/j.1365-313x.1999.00496.x 10417719

[B7] Gernand D Demidov D Houben A The temporal and spatial pattern of histone H3 phosphorylation at serine 28 and serine 10 is similar in plants but differs between mono- and polycentric chromosomes Cytogenet Genome Res 2003 101 2 172 176 10.1159/000074175 14610360

[B8] Kurihara D Matsunaga S Kawabe A Fujimoto S Noda M Uchiyama S Fukui K Aurora kinase is required for chromosome segregation in tobacco BY-2 cells Plant J 2006 48 4 572 580 10.1111/j.1365-313X.2006.02893.x 17087760

[B9] Zhang X Li X Marshall JB Zhong CX Dawe RK Phosphoserines on maize CENTROMERIC HISTONE H3 and histone H3 demarcate the centromere and pericentromere during chromosome segregation Plant Cell 2005 17 2 572 583 10.1105/tpc.104.028522 15659628 PMC548827

[B10] Demidov D Van Damme D Geelen D Blattner FR Houben A Identification and dynamics of two classes of aurora-like kinases in Arabidopsis and other plants Plant Cell 2005 17 3 836 848 10.1105/tpc.104.029710 15722465 PMC1069702

[B11] Kawabe A Matsunaga S Nakagawa K Kurihara D Yoneda A Hasezawa S Uchiyama S Fukui K Characterization of plant Aurora kinases during mitosis Plant Mol Biol 2005 58 1 1 13 10.1007/s11103-005-3454-x 16028112

[B12] Demidov D Hesse S Tewes A Rutten T Fuchs J Ashtiyani RK Lein S Fischer A Reuter G Houben A Aurora1 phosphorylation activity on histone H3 and its cross-talk with other post-translational histone modifications in Arabidopsis Plant J 2009 59 2 221 230 10.1111/j.1365-313X.2009.03861.x 19582900

[B13] Dai J Sultan S Taylor SS Higgins JM The kinase haspin is required for mitotic histone H3 Thr 3 phosphorylation and normal metaphase chromosome alignment Genes Dev 2005 19 4 472 488 10.1101/gad.1267105 15681610 PMC548948

[B14] Preuss U Landsberg G Scheidtmann KH Novel mitosis-specific phosphorylation of histone H3 at Thr11 mediated by Dlk/ZIP kinase Nucleic Acids Res 2003 31 3 878 885 10.1093/nar/gkg176 12560483 PMC149197

[B15] Houben A Demidov D Rutten T Scheidtmann KH Novel phosphorylation of histone H3 at threonine 11 that temporally correlates with condensation of mitotic and meiotic chromosomes in plant cells Cytogenet Genome Res 2005 109 1-3 148 155 10.1159/000082394 15753571

[B16] Caperta AD Rosa M Delgado M Karimi R Demidov D Viegas W Houben A Distribution patterns of phosphorylated Thr 3 and Thr 32 of histone H3 in plant mitosis and meiosis Cytogenet Genome Res 2008 122 1 73 79 10.1159/000151319 18931489

[B17] Tanaka H Yoshimura Y Nishina Y Nozaki M Nojima H Nishimune Y Isolation and characterization of cDNA clones specifically expressed in testicular germ cells FEBS Lett 1994 355 1 4 10 10.1016/0014-5793(94)01155-9 7957958

[B18] Tanaka H Yoshimura Y Nozaki M Yomogida K Tsuchida J Tosaka Y Habu T Nakanishi T Okada M Nojima H Identification and characterization of a haploid germ cell-specific nuclear protein kinase (Haspin) in spermatid nuclei and its effects on somatic cells J Biol Chem 1999 274 24 17049 17057 10.1074/jbc.274.24.17049 10358056

[B19] Higgins JM The Haspin gene: location in an intron of the integrin alphaE gene, associated transcription of an integrin alphaE-derived RNA and expression in diploid as well as haploid cells Gene 2001 267 1 55 69 10.1016/S0378-1119(01)00387-0 11311556

[B20] Dai J Sullivan BA Higgins JM Regulation of mitotic chromosome cohesion by Haspin and Aurora B Dev Cell 2006 11 5 741 750 10.1016/j.devcel.2006.09.018 17084365

[B21] Dai J Kateneva AV Higgins JM Studies of haspin-depleted cells reveal that spindle-pole integrity in mitosis requires chromosome cohesion J Cell Sci 2009 122 Pt 22 4168 4176 19910498 10.1242/jcs.054122 PMC2776503

[B22] Yamagishi Y Honda T Tanno Y Watanabe Y Two histone marks establish the inner centromere and chromosome bi-orientation Science 2010 330 6001 239 243 10.1126/science.1194498 20929775

[B23] Wang F Dai J Daum JR Niedzialkowska E Banerjee B Stukenberg PT Gorbsky GJ Higgins JM Histone H3 Thr-3 phosphorylation by Haspin positions Aurora B at centromeres in mitosis Science 2010 330 6001 231 235 10.1126/science.1189435 20705812 PMC2967368

[B24] Kelly AE Ghenoiu C Xue JZ Zierhut C Kimura H Funabiki H Survivin reads phosphorylated histone H3 threonine 3 to activate the mitotic kinase Aurora B Science 2010 330 6001 235 239 10.1126/science.1189505 20705815 PMC3177562

[B25] Higgins JM Structure, function and evolution of haspin and haspin-related proteins, a distinctive group of eukaryotic protein kinases Cell Mol Life Sci 2003 60 3 446 462 10.1007/s000180300038 12737306 PMC11138542

[B26] Phytozome v 6.0 http://www.phytozome.net/

[B27] Higgins JM Haspin-like proteins: a new family of evolutionarily conserved putative eukaryotic protein kinases Protein Sci 2001 10 8 1677 1684 10.1110/ps.49901 11468364 PMC2374090

[B28] Eswaran J Patnaik D Filippakopoulos P Wang F Stein RL Murray JW Higgins JM Knapp S Structure and functional characterization of the atypical human kinase haspin Proc Natl Acad Sci USA 2009 106 48 20198 20203 10.1073/pnas.0901989106 19918057 PMC2777956

[B29] Villa F Capasso P Tortorici M Forneris F de Marco A Mattevi A Musacchio A Crystal structure of the catalytic domain of Haspin, an atypical kinase implicated in chromatin organization Proc Natl Acad Sci USA 2009 106 48 20204 20209 10.1073/pnas.0908485106 19918049 PMC2777964

[B30] Zimmermann P Hirsch-Hoffmann M Hennig L Gruissem W GENEVESTIGATOR. Arabidopsis microarray database and analysis toolbox Plant Physiol 2004 136 1 2621 2632 10.1104/pp.104.046367 15375207 PMC523327

[B31] Menges M Hennig L Gruissem W Murray JA Cell cycle-regulated gene expression in Arabidopsis J Biol Chem 2002 277 44 41987 42002 10.1074/jbc.M207570200 12169696

[B32] Guo HS Fei JF Xie Q Chua NH A chemical-regulated inducible RNAi system in plants Plant J 2003 34 3 383 392 10.1046/j.1365-313X.2003.01723.x 12713544

[B33] Ohashi S Sakashita G Ban R Nagasawa M Matsuzaki H Murata Y Taniguchi H Shima H Furukawa K Urano T Phospho-regulation of human protein kinase Aurora-A: analysis using anti-phospho-Thr288 monoclonal antibodies Oncogene 2006 25 59 7691 7702 10.1038/sj.onc.1209754 16785988

[B34] Gurley LR Walters RA Tobey RA Sequential phsophorylation of histone subfractions in the Chinese hamster cell cycle J Biol Chem 1975 250 10 3936 3944 1168641

[B35] Johansen KM Johansen J Regulation of chromatin structure by histone H3S10 phosphorylation Chromosome Res 2006 14 4 393 404 10.1007/s10577-006-1063-4 16821135

[B36] Dai J Higgins JM Haspin: a mitotic histone kinase required for metaphase chromosome alignment Cell Cycle 2005 4 5 665 668 10.4161/cc.4.5.1683 15846065

[B37] Matsuoka K Demura T Galis I Horiguchi T Sasaki M Tashiro G Fukuda H A comprehensive gene expression analysis toward the understanding of growth and differentiation of tobacco BY-2 cells Plant Cell Physiol 2004 45 9 1280 1289 10.1093/pcp/pch155 15509851

[B38] Kazusa Tomato SBM Database http://www.kazusa.or.jp/tomato/

[B39] Verma DP Cytokinesis and Building of the Cell Plate in Plants Annu Rev Plant Physiol Plant Mol Biol 2001 52 751 784 10.1146/annurev.arplant.52.1.751 11337415

[B40] Sedbrook JC Kaloriti D Microtubules, MAPs and plant directional cell expansion Trends Plant Sci 2008 13 6 303 310 10.1016/j.tplants.2008.04.002 18467155

[B41] Houben A Demidov D Caperta AD Karimi R Agueci F Vlasenko L Phosphorylation of histone H3 in plants--a dynamic affair Biochim Biophys Acta 2007 1769 5-6 308 315 17320987 10.1016/j.bbaexp.2007.01.002

[B42] Kurihara D Matsunaga S Uchiyama S Fukui K Live cell imaging reveals plant aurora kinase has dual roles during mitosis Plant Cell Physiol 2008 49 8 1256 1261 10.1093/pcp/pcn098 18593743

[B43] Seki M Narusaka M Kamiya A Ishida J Satou M Sakurai T Nakajima M Enju A Akiyama K Oono Y Functional annotation of a full-length Arabidopsis cDNA collection Science 2002 296 5565 141 145 10.1126/science.1071006 11910074

[B44] Kurihara D Kawabe A Matsunaga S Nakagawa K Fujimoto S Uchiyama S Fukui K Characterization of a splicing variant of plant Aurora kinase Plant Cell Physiol 2007 10.1093/pcp/pcl064 17202181

[B45] Fujimoto S Matsunaga S Yonemura M Uchiyama S Azuma T Fukui K Identification of a novel plant MAR DNA binding protein localized on chromosomal surfaces Plant Mol Biol 2004 56 2 225 239 10.1007/s11103-004-3249-5 15604740

[B46] Nagai T Ibata K Park ES Kubota M Mikoshiba K Miyawaki A A variant of yellow fluorescent protein with fast and efficient maturation for cell-biological applications Nat Biotechnol 2002 20 1 87 90 10.1038/nbt0102-87 11753368

[B47] Kumagai F Yoneda A Tomida T Sano T Nagata T Hasezawa S Fate of nascent microtubules organized at the M/G1 interface, as visualized by synchronized tobacco BY-2 cells stably expressing GFP-tubulin: time-sequence observations of the reorganization of cortical microtubules in living plant cells Plant Cell Physiol 2001 42 7 723 732 10.1093/pcp/pce091 11479379

[B48] Clough SJ Bent AF Floral dip: a simplified method for Agrobacterium-mediated transformation of Arabidopsis thaliana Plant J 1998 16 6 735 743 10.1046/j.1365-313x.1998.00343.x 10069079

[B49] Truernit E Bauby H Dubreucq B Grandjean O Runions J Barthelemy J Palauqui JC High-resolution whole-mount imaging of three-dimensional tissue organization and gene expression enables the study of Phloem development and structure in Arabidopsis Plant Cell 2008 20 6 1494 1503 10.1105/tpc.107.056069 18523061 PMC2483377

