# Supplementary figures and images for: Identification and characterization of plant Haspin kinase as a histone H3 threonine kinase
Source: BMC Plant Biol. 2011 Apr 28;11:73. doi: 10.1186/1471-2229-11-73 (PMC3113928; doi:10.1186/1471-2229-11-73)

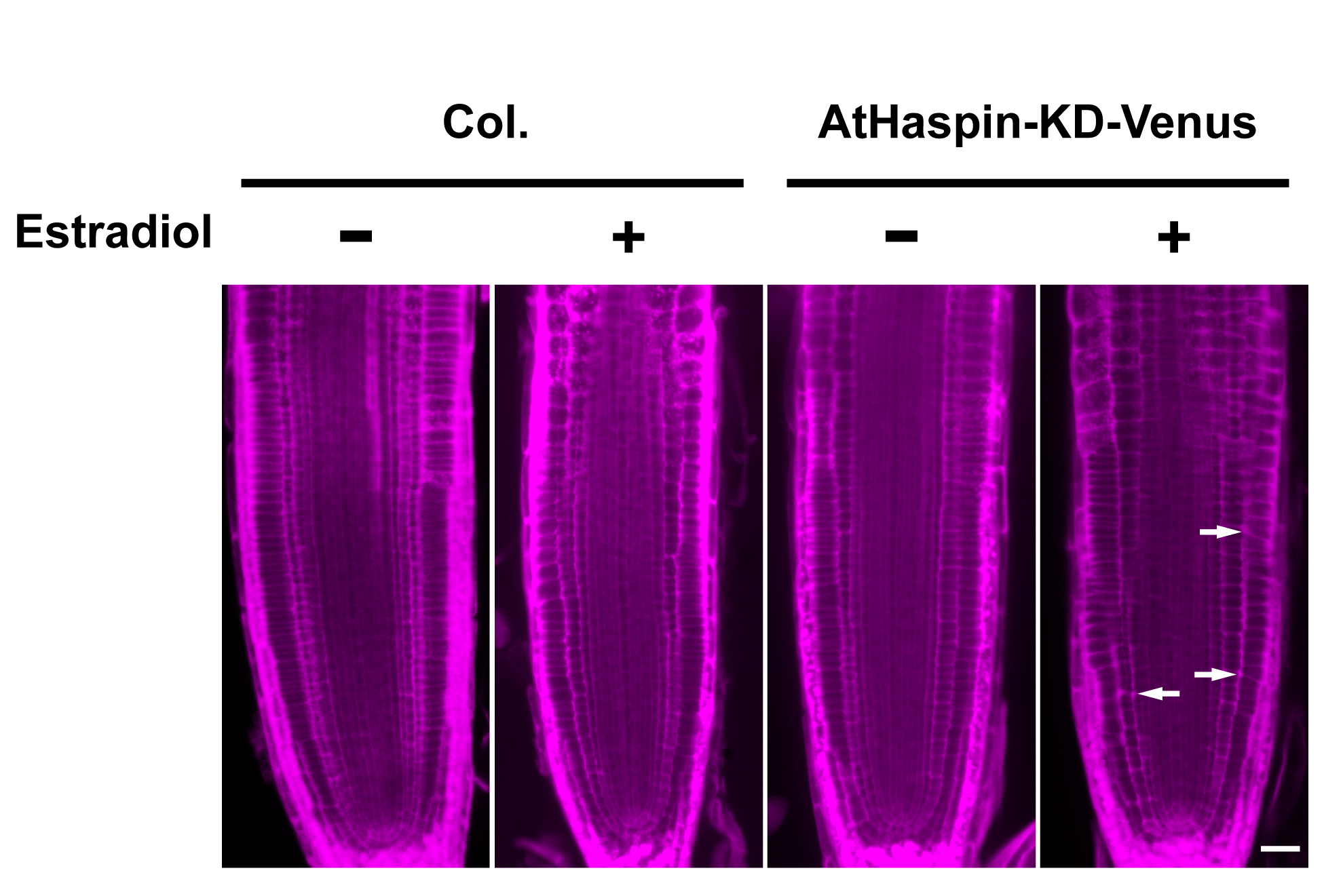

Supplement: Additional file 5 — Abnormality of cell orientation in AtHaspin-KD plants. At 6 days after imbibition, Col. plants and transformants with inducible AtHaspin-KD-Venus vectors with or without induction were analyzed using the mPS-PI method. In AtHaspin-KD plants, arrows indicate abnormalities in orientations of cell walls between transverse neighboring cells. Scale bar: 50 μm. [file 1471-2229-11-73-S5.TIFF]
